# Occupational therapy assessment and interventions for young autistic children in South Africa

**DOI:** 10.4314/ahs.v23i1.77

**Published:** 2023-03

**Authors:** Aneesa Moosa, Thavanesi Gurayah, Saira Banu Karim, Pragashnie Govender

**Affiliations:** 1 Occupational Therapist in Private Practice, Durban, South Africa; 2 Discipline of Occupational Therapy, School of Health Sciences University of KwaZulu Natal, South Africa; 3 Discipline of Speech Language Therapy, School of Health Sciences University of KwaZulu Natal, South Africa

**Keywords:** Occupational therapy, occupational therapy assessment, occupational therapy intervention, autism spectrum disorder, autistic children, South African practice

## Abstract

**Background:**

Occupational Therapy is among the top interventions for autistic children, hence the need for equitable and effective services in the public and private health and education sectors. Ongoing research into the therapies for autism spectrum disorders in different contexts is also required.

**Objectives:**

To explore and describe occupational therapists' assessment and intervention for autistic children in South Africa.

**Methods:**

A descriptive qualitative research design using semi-structured interviews to gather data from purposively recruited OTs (n=20). Data were audio-recorded, transcribed and analysed thematically, and compared across three sectors public health, special needs schools and private practice.

**Results:**

South African practice across all three sectors was similar to international patterns of informal play-based assessment, sensory processing and Ayres Sensory Integration (ASI®) treatment. Developmental frameworks guided specific approaches. Strong team collaboration was present across sectors, with some transdisciplinary teamwork and co-treatment. Undergraduate and postgraduate training opportunities were, however limited.

**Conclusions:**

Occupational therapy assessments had diagnostic value. Informal tools such as developmental checklists were found to have clinical utility, whilst standardised tools were most commonly used to assess sensory processing and visual perception. Recommendations included incorporating ASI® into undergraduate curricula and postgraduate training opportunities with multidisciplinary input to develop ASD professionals in South Africa. It is imperative to advocate for services in under-resourced rural areas and marginalised communities that lack financial and social resources. Occupational therapists need to find new ways of working collaboratively across sectors to ensure effective and comprehensive services in public health and special schools.

## Introduction

Autism Spectrum Disorders (ASD) affects one in every 68 children in the United States of America^1^ and approximately one in every 100 children in South Africa^2^. With the burgeoning incidence of ASD worldwide, the demand for all professional services is expected to grow to promote the participation of autistic persons in all facets of society. In most settings, the multidisciplinary team (MDT) would include occupational therapists, who have a role in assessing and treating autistic children and adults^3^,^4^.

Due to the increase in research outputs across disciplines, to explore and describe the current assessment and treatment strategies, occupational therapists should keep abreast of the contemporary and most efficacious interventions for autistic children. They need to contribute contextually relevant research evidence from various geographical contexts is also required. The demand for all professional services is expected to grow to promote the participation of autistic persons in all facets of society. Autistic children have numerous occupational and performance impairments that limit their participation in the home, school and community contexts. Early identification of autistic children, and effective intervention, have become key to reducing the burden of disability on the child and family^5^–^7^. Most programmes for young autistic children, including occupational therapy, incorporate developmental approaches. The core features of autistic children include impairments in communication and social interaction, stereotypical movement and behaviours, and sensory challenges^8^. In occupational therapy, the emphasis is on the facilitation of the child's sensory processing, sensorimotor development, social-behavioural performance, self-care and participation in play^9^.

Most programmes for young autistic children, including occupational therapy incorporate developmental approaches. Intervention is play-based, and uses peers and the child's strengths in therapy^10^. Occupational therapists utilise several frameworks, the most popular being Ayres Sensory Integration (ASI®), Developmental, Individual Difference, Relationship-based Floortime (DIR®/Floortime ™), Augmentative and Alternative Communication (AAC) and behavioural models, indicating an eclectic approach to intervention^11^,^12^,^13^,^14^.

The rationale for this study follows from several perspectives. From a research perspective, only five published South African studies on occupational therapy and ASD were found in a database search. These dealt with aspects of service provision^15^, parent-child sensory compatibility^16^, the efficacy of sensory integration intervention^17^ and parents' perspectives on experiences of ASD^18^,^19^. None describing the practice patterns of South African occupational therapists across sectors of private practice, public health and special needs schooling were found. Secondly, the benefits of occupational therapy in management programmes for autistic children can be immense in terms of the occupational therapists' expertise in treating varying sensory profiles and gross and fine motor impairments. The patient profile and contextual factors (a unique post-apartheid health, education, socioeconomic and cultural context) are often a challenge to occupational therapists. In South Africa, the majority of the population is dependent on public health services, for which they have to travel a distance at an unaffordable cost. Accessibility to culturally and linguistically relevant assessment and management resources is also limited for occupational therapists and their autistic clientele. The aforementioned factors require management strategies that support child development and progress, with due cognisance of the challenges. This study explored and describes occupational therapists' assessment and intervention for autistic children in South Africa.

## Methods

### Research Team and Reflexivity

The primary author, an occupational therapist, was the study lead and responsible for the data collection in the study and completed the study as part of postgraduate credentialing. All authors are female health professionals who have experience in the field of ASD. A collegial/professional relationship existed between the participants, and the primary author, who was actively involved in providing interventions for autistic children at the time of the study. The primary author entered decisions and feelings and preconceptions into a reflexive diary throughout the study to ensure suspension of judgements. Peer debriefing with the co-authors assisted in maintaining a level of objectivity in the study and in reducing potential researcher bias.^20^,^21^

### Study Design

**Theoretical Orientation:**

An explorative qualitative research design^20^, within an interpretive paradigm, was conducted using individual interviews.

### Participant Selection

Therapists were invited electronically via the Occupational Therapy Association of South Africa (OTASA). Purposive criterion sampling^21^ was utilised to recruit participants who met the following criteria: (i) a current registration with the health professions board, (ii) a minimum of two years' experience working with autistic children aged two to 12 years and (iii) any race, gender or cultural background. Twenty occupational therapists were recruited into the study based on their experience and location in one of three sectors, namely, education, public health, or private health. There were no non-participants present in the interviews. Essential characteristics of the sample are provided.

### Setting

The study was located in South Africa across nine provinces. Interviews were conducted at the most convenient venue identified by the occupational therapists.

### Data Collection

The data were gathered using audio-recorded semi-structured individual interviews (n=16) and two dyad interviews (n=4). The interviews spanned approximately 60 to 120 minutes. The interview schedule was guided but not based directly on the surveys by Watling et al.^22^ and Case-Smith & Miller^23^, which described practice patterns of occupational therapists in the USA. Questions and probes around assessment, treatment, indirect therapy, education and training, and challenges to South African families were included in the schedule. No repeat interviews occurred. Data were collected until redundancy was achieved, as data saturation would have been challenging to achieve given the diversity of the group. Member checking strategies occurred at the end of the interviews.

### Data Analysis and Findings

Data were analysed thematically^24^ following a review of the transcripts for veracity by the primary author and verified by the co-authors. Initial codes and categories were developed into sub-themes using deductive reasoning according to the interview schedule. Themes are reported descriptively and are augmented by verbatim quotes.

### Trustworthiness

The use of a pilot study^25^ to test the interview schedule, interview etiquette of the interviewer, and practice analysis of the data ensured credibility of the process. Following the pilot, interview questions were revised to exclude a section on discharge patterns and the sequence of questions were reorganised for improved coherence. During the interviews, frequent member checks occurred for clarification and to ensure information was captured correctly. Credibility was further enhanced by peer debriefing, the use of field notes, and immersion in the data through the analysis phase^21^,^24^. The narrative descriptions of the data with verbatim quotes and the use of purposive sampling supported transferability^21^,^24^.

### Ethical considerations

Ethical approval was obtained from the Humanities and Social Sciences Research Ethics Committee at the University of KwaZulu-Natal (HSS/0060/012M). Gatekeeper permission was obtained from the Departments of Education and Health to access participants. Written informed consent was obtained from all participants and confidentiality of their identities assured by the use of pseudonyms.

### Findings

All 20 therapists were female. Ages ranged from 26 to 57 years (M=41 years). Their clinical practice experience with ASD ranged from two to 27 years (M= 14.9 years). Of these, 19 of the participants had completed additional coursework. These included ASI®(n=12), NDT (n=5), Makaton signing (n=2), Picture Exchange Communication System (PECS) (n=1), DIR®/Floortime™ Model (n=6), Therapeutic Listening/Tomatis Training (n=4). In terms of geographical location, eight therapists were from the Western Cape province, seven were from Gauteng province, and five were from KwaZulu-Natal province. Practice settings included private practice (n=8), special needs schools (n=6) and public hospitals (n=6).

The findings are presented according to four emergent themes, i.e. (i) assessment, (ii) intervention, (iii) education and training, and (iv) challenges to South African families and these are compared across the three sectors.

### Theme 1: Assessment

Half of the occupational therapists (n=10) worked in interdisciplinary teams or in an MDT. Discipline-specific assessments included occupational therapy, speech-language therapy, physiotherapy and educational evaluations. Public hospital occupational therapists contributed routinely to ASD diagnosis. They were often the first team members to assess a child, alerting doctors to a suspected diagnosis. Occupational therapists in private practice also referred children to paediatricians for a diagnosis. Only two hospital-based occupational therapists participated in diagnostic evaluations in MDT and outreach team clinics.

In hospitals and private practices, assessments occur over more than one session. Initial interaction in the evaluation was non-invasive, on the child's terms, and with a gradual introduction to a new physical and social environment. Sensory stimuli in the environment were decreased to prevent sensory overload.

Jessica: *“I treated a little boy on his father's feet for about three sessions... the relationship is paramount, and those first three, four sessions is where you make or break it...if the child trusts you, you can really make them go where you want to...”*

Informal assessment required minimal equipment, sufficient knowledge of ASD, excellent observation skills, and engagement in playful interaction with the child. Therapists used various toys and equipment to assess sensory processing, conceptual, cognitive and gross and fine motor skills. All but one facility had access to gross-motor rooms with a platform swing, hammock, mini trampoline and large gym balls.

Carla: *“...assessment material, so it's a variety of different types of toys, so things that feel squashy or your pretend play toys, cause and effect type of toys and – a whole variety of toys where I can interact with the child...and take them to a table top where I try and look at their fine motor ... I look at their motor development throughout this, how they're planning...bring out a form board...ball skills”*

All therapists (n=20) conducted a detailed caregiver interview to gather information about the background history, child's sensory preferences, instrumental activities of daily living, behaviour in different environments, and occupational roles of the child, including play. Therapists reported pacing their assessment based on their observation of the child, to prevent the pressure of an assessment context on the child.

Jessica: *“definitely if there's any indication of autistic, I don't put pressure of an assessment at all”*

All therapists (n=20) conducted informal play-based, observational assessment to provide a holistic profile of a child's strengths and impairments. Skilled observation by the occupational therapist required an awareness of the various performance components, developmental norms and ASD characteristics. Therapists felt competent to conduct observations based on their experience and training. The occupational therapists used three categories of non-standardised instruments, including developmental checklists, a play scale and a parent-child interaction scale. Play as an occupation was assessed in private practice and hospitals, possibly due to the younger ages of children, while academic issues were a priority in special needs schools. The value of informal play-based assessments over standardised testing for ASD was evident across all sectors.

All occupational therapists across sectors agreed that standardised tests in young autistic populations had limited clinical utility. Standardised tests were used to measure sensory processing (most commonly used), sensory-motor function and visual perceptual skills, as detailed in Table 1.

**Table 1:** Assessment Instruments (non-standardised and standardised) (n=20)

ASSESSMENT		n
**Non-standardised** **Tests/Instruments**	**Developmental Checklists**	
	Statewise Autism Resources and Training (START)^26^	3
	WITS Developmental Profile^27^	3
	Rita Edwards Developmental Checklists^28^	2
	Accelerate Pre-school Enrichment Programmes^29^	2
	Carla Grobler's Developmental Checklist^30^	1
	**Play Scales**	
	Knox Play Scale^31^	1
	Functional Emotional Assessment Scale (FEAS)^32^	2
**Standardised** **Instruments/Tests**	**ASD-specific Assessments**	
	Autism Diagnostic Observation Schedule (ADOS-2)^33^	2
	Childhood Autism Rating Scale (CARS-2)^34^	2
	Psycho-Educational Profile (PEP)^35^	1
	**Sensory Processing Profiles**	
	Sensory Profile^36^	14
	Sensory Profile-School Companion^36^	2
	Infant/Toddler Symptom Checklist (ITSC)^37^	2
	**Tests of Sensory Integration**	
	Sensory Integration and Praxis Test (SIPT)^38^	3
	Test of Sensory Function in Infants^39^	2
	Test of Sensory Integration^40^	2
	**Test of Sensory Motor Function**	
	Miller Assessment for Preschoolers^41^	4
	Miller Function & Participation Scales (M-FUN-S)^42^, ^43^	1
	**Visual Perceptual and Cognitive Tests**	
	Beery-Buktenika Developmental Test of Visual- Motor Integration (VMI)^44^	13
	Developmental Test of Visual Perception (DTVP-2)^45^	13
	Test of Visual Perceptual Skills-revised (TVPS-R)46	13

The value of standardised tests was most useful in private practice settings for assessing sensory processing, sensory integration and motor skills in early intervention with visual perceptual testing typically occurring prior to school placement.

Queen:*” ...they are referred at age of 3 and then usually they struggle with most things...after they had maybe two years of therapy, I will actually do this (DTVP-2, Beery Developmental tests), maybe a year before they actually have to be placed.”*

Even then, the use of standardised tests with autistic children was most often used in an adapted form. The SIPT38 was used by only three of twelve certified occupational therapists, all in private practice. Clinical utility of standardised testing was limited due to factors such as the length of the test and time to administer, suitability of the test for autistic children, contextual issues such as private versus public sector, language of the test for non-English language speakers, attention span, level of cognitive functioning, receptive language abilities of the child and the prohibitive costs of standardised tests.

### Theme 2: Intervention

In direct intervention, the typical service provision models used were individual sessions, small group therapy sessions, intensive block therapy and co-treatment. In public health, transport costs hampered intensive block therapy due to services located outside of communities.

Queen: *“the parents financially they actually can't afford (it). Remember we're not a hospital that is actually close to the community...so to come here a number of them have to use two taxis, if not three.*

Intensive blocks of therapy in private health were hampered by prohibitive financial costs to parents. Special needs schools had limited resources to provide more frequent services to the level that was preferred. Special needs schools typically run a combination of groups (for gross motor activities) and individual sessions, with pupils receiving either or both forms of therapy based on need or priority. All school therapists indicated the need to do more individual intervention, but were constrained by time and human resources. Thus, they prioritised children with higher sensory needs and behavioural issues.

Serena: *“You're going to prioritise a child with severe sensory and behavioural issues over a child who is functioning well but they are not writing...the ones who are disruptive in the class, we unfortunately, do prioritise them.”*

Class-based interventions that moved therapy into the classroom, in line with inclusionary principles, was in the early stages of practice in some special needs schools.

Charlize: *“We setting up a lot of programmes, it's the plan ...that we will do as little as possible individual therapy but more class-based interventions”*

Typically, group therapy occurs for visual perceptual, fine and gross motor problems, in the classroom in addition to individual sessions in occupational therapy. Class based intervention has been hailed as a solution to service provision dilemmas due to high pupil to therapy ratios. Aside from the observable benefits of skills sharing and transference to teachers, the benefit of contextual practice and integration of occupational therapy into the classroom for autistic pupils contributes to the generalisation of skills. The full value of collaborative class-based therapy approaches was not used in Special Needs Schools (SNS). Occupational therapists often collaborated with speech-language therapists in the co-assessment and/or treatment of a child in public health settings, but co-treatment was limited in SNS where MDT modes of working is most prevalent. Co-treatment assisted with sensory regulation for children who were difficult to manage and occurred at the start of therapy when sensory issues were most prominent.

Rebecca:*” they (teachers) just integrate principles and ideas- you walk into the classrooms and then it might look just like a therapy (sessions)...teachers that have been so therapised...they also expect... even more new things. As OT that's a huge role, is thinking new ways, innovative, creation – because therapy s been here for so long.”*

ASI® developmental and behavioural frameworks, neurodevelopmental therapy, Vona du Toit Model of Creative Ability (VdTMOCA)47, DIR®/Floortime32, AAC and social stories were the theoretical approaches used by occupational therapists in their management of autistic children with all therapists agreeing that ASI® is an essential and primary frame of reference. VdTMOCA47, a uniquely South African model, was being used by three occupational therapists across all three sectors. VdTMOCA47 applicability to assessment and intervention for ASD populations is a novel avenue for further research. The dosage or frequency of intervention has implications for success in early intervention and continues to be a challenge across all three sectors. Occupational therapists in private practice provided services once or twice a week for thirty to forty-five minutes, reduced to once fortnightly with significant progress, which was financially challenging for parents. Children were seen once fortnightly or monthly for between twenty and sixty minutes in hospitals, where dosage was impacted negatively due to the financial implications of travel, even when utilising intensive block scheduling versus long term weekly sessions. One hospital developed an outreach distance home programme which was monitored every three months. Creative service provision options may be possible in SNS but less likely to succeed in public health due to the financial implications of travel costs. This also raises the question of the viability of ASI® intervention for public health and SNS settings in South Africa, which has a higher demand for services.

**Figure 1:**
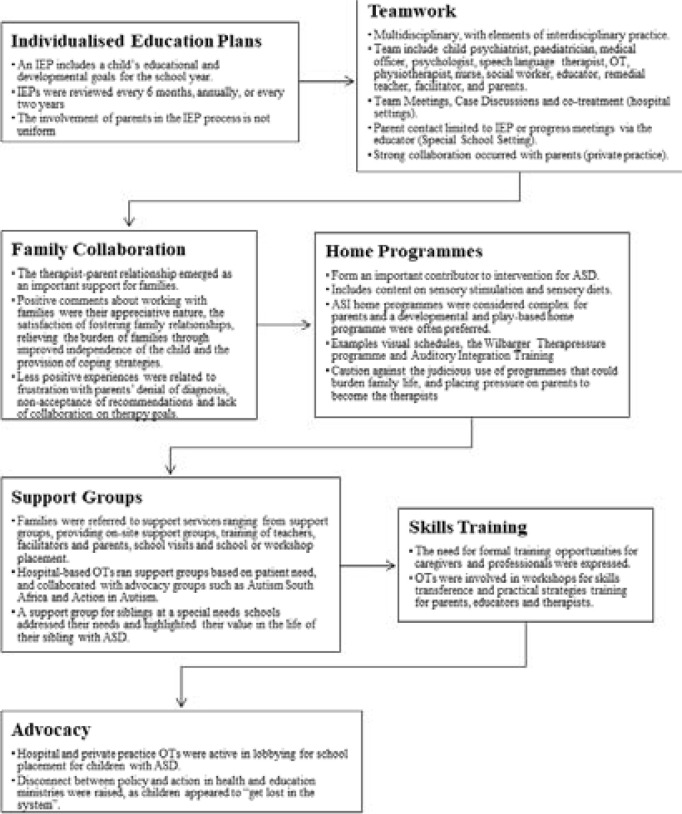
Indirect Interventions reported by the occupational therapists in this study

The indirect intervention (n=20) included individualised education plans (IEP) where the teamwork, family collaboration, home programmes, support groups, skills training and advocacy were possible (Figure 1). A lack of parental involvement in most SNS settings was however a concern.

### Theme 3: Education and training for occupational therapists

Generally, occupational therapists felt that their undergraduate training did not adequately prepare them for working with autistic children. More recently qualified occupational therapists however had exposure to ASD at undergraduate level. Notwithstanding this, entry into the field of ASD was daunting for new graduates. Discouraging comments such as ASD is a “specialised area” that requires specialist skills made occupational therapists reluctant to treat autistic children if they were not ASI® trained. Eight occupational therapists suggested the inclusion of sensory-based intervention theory on sensory processing and modulation at an undergraduate level would equip new graduates to work with autistic children and developmental conditions across the lifespan, thus demystifying the condition for occupational therapists, and reducing negative perceptions around treating ASD. Generally occupational therapists expressed that postgraduate study, with MDT input, in the field of ASD would be beneficial. Some occupational therapists (n=4) questioned whether a postgraduate degree would necessarily make one a skilled clinician, without practical application to develop clinical skills and expertise; whilst others (n=3) proposed a professional support group and mentorship programme.

### Theme 4: Challenges to autistic children and their families in South Africa

Awareness of ASD, availability of facilities and services, and social issues impacted autistic children and their families. The general public indicated a general lack of awareness of ASD as a developmental disability (n=9), with stigma and cultural misconceptions about ASD prevailing. As Serena suggested, “the stigma...they get called bewitched”. The value of the sensory profile as an assessment tool in dispelling cultural myths surrounding autistic behaviours and reframing parent perspectives is significant. There were also concerns that children at risk for developmental disorders were not consistently referred by general practitioners and nurses, possibly due to the limited knowledge of professionals.

A lack of appropriate and available schooling facilities for autistic children at different levels of functioning was cited as a significant challenge (n=17). Occupational therapists were concerned about insufficient access to therapy attributed to transport costs in the public health sector, limited practical training for parents and professional staff involved with ASD, human resource challenges, a shortage of qualified professionals, and a lack of ASD specialists.

## Discussion

South African occupational therapy practice in the field of ASD is similar to international practice across sectors despite local challenges. The use of the VdTMOCA^47^, is possibly the only unique SA practice. Occupational therapists formed part of interdisciplinary and MDT teams and had a role in assessing and treating autistic children^3^,^4^. The assessment patterns demonstrated a preference for informal play-based skilled observation using developmental checklists and play scales, many of which were developed in South Africa. The unique focus of occupational therapy assessments was sensory-motor processing, which had diagnostic value in some settings. All occupational therapists concurred that the role and value of standardised tests were limited, and occupational therapy specific standardised tests were often used in an adapted format. ASD-specific standardised tests such as the ADOS-2^33^, CARS-2^34^ and the PEP^35^ were used by some occupational therapists in confirming the ASD diagnosis.

The Sensory Profile^36^ was the most commonly used standardised tool. Recently, a study on the clinical utility of the Sensory Profile, was published on its recommended use and modifications for such a culturally diverse population^48^. The SIPT^38^ was used infrequently to assess sensory integration due to the unsuitability of the test (length, language-based instructions and complexity) for autistic children. The Miller Assessment for Preschoolers^41^ was more commonly used to assess sensory-motor function than the M-FUN-S^42^,^43^. Visual perceptual and cognitive tests were used to screen for specific learning problems in autistic children. Standardised testing was most utilised in private practice and school settings.

The focus of ASI® intervention was sensory modulation which appears to be in keeping with international trends^9^. The sensory profile and sensory modulation were the third most commonly requested intervention by caregivers^49^. Clinical practice of ASI® for ASD lacks clarity in terms of fidelity due to the need for structure for ASD intervention. ASI® and DIR®/Floortime™^14^ were often practised together. The family-focussed practice was evident in private and public health sectors, despite the resource constraints of the latter. Direct individual therapy was the predominant intervention model across all sectors, with less emphasis on class-based interventions, despite the challenge of low staff to high client ratio, the intensity of treatment required, and the necessity for contextual learning in ASD. Task shifting that moved some of the occupational therapists' responsibilities to the teacher was practiced at some schools, and is an inclusionary principle. All participants conducted a detailed caregiver interview, including roles, habits and rituals of the child, as well as the occupational balance of the caregiver^50^, which confirms the holistic nature of the assessment and intervention despite the overt sensory-motor focus.

There appeared to be a lack of postgraduate educational and training opportunities for ASD in South Africa. Many participants did not feel equipped to treat autistic children with the limited exposure at university, which was especially daunting for new graduates. Many participants felt that sensory-based interventions should be incorporated at the undergraduate level, as understanding sensory processing and modulation elements had benefits for working with clients with ASD and other developmental disorders across the lifespan.

Families experienced stigma and cultural misconceptions about ASD, which were attributed to the general public's lack of awareness of ASD. As confirmed by other studies, occupational therapists raised the lack of appropriate schooling facilities and the social and financial strains experienced by parents. These are themes that have emerged in other studies by occupational therapists that considered the parent perspective in the care of children diagnosed with ASD^15^,^18^,^19^.

## Limitations

This study provided valuable insights into the assessment and intervention practice of occupational therapists in the field of ASD, which is lacking in the available literature. Positioned as a national study, a limitation was that participants from only three of nine provinces in the country agreed to participate. Furthermore, the research was confined to the three major cities, with no data from rural areas. Notwithstanding this, three practice areas, namely, public health, private health and education sector participants, formed the sample, which allowed comparison across sectors. All therapists were female. Future studies should consider a more diverse group of therapists across practice contexts and less-resourced provinces such as the Eastern Cape and Mpumalanga, and should include rural areas, to illuminate the practice of occupational therapists in these areas. Comparison to current best practices may also be useful.

## Conclusion

Assessment and intervention practice patterns of occupational therapists in South Africa across all three sectors were similar to international practice patterns despite local challenges. Assessment was primarily informal and play based, having cross-cultural utility. Sensory processing and sensory motor function were a unique occupational therapy assessment focus. Standardised assessments had a limited role primarily in academic settings. Occupational therapy intervention was informed by developmental frameworks with ASI® and sensory-based interventions an essential and primary frame of reference, due to the significant prevalence off sensory processing challenges in autistic children. A recommendation for sensory-based interventions to be included in undergraduate curricula may be beneficial, as most occupational therapists felt daunted and ill-equipped for working in the field. The suggestion of ASD-specific postgraduate courses with multidisciplinary input may also assist in addressing the shortage of ASD professionals in South Africa. The challenge in providing comprehensive services in under-resourced rural areas, and to marginalised communities without financial and social resources remains. The need for greater consultation and indirect services to address the demand for services at school level is a policy level recommendation that remains to be realised at the ground level.
